# Supplementary material for: Importance and implementation of safe nursing behaviors in nursing students’ clinical practice: Importance–Performance Analysis (IPA), the borich needs assessment model, and the locus for focus model
Source: PLoS One. 2026 Mar 30;21(3):e0344741. doi: 10.1371/journal.pone.0344741 (PMC13035157; doi:10.1371/journal.pone.0344741)
Supplement: S1 Table — (DOCX) [file pone.0344741.s001.docx]

**Supplementary Table S1. Internal Consistency Reliability (Cronbach’s α) for Importance and Performance Scales**

| Variables | Items | Cronbach’s α  (Importance) | Cronbach’s α (Performance) |
| --- | --- | --- | --- |
| Total scale | 28 | 0.90 | 0.92 |
| I. Infection Prevention | 13 | 0.83 | 0.83 |
| II. Musculoskeletal Injury Prevention | 3 | 0.55 | 0.69 |
| III. Chemical Hazard Prevention | 3 | 0.83 | 0.72 |
| IV. Psychological Injury Prevention | 9 | 0.77 | 0.84 |
