# Supplementary material for: Importance and implementation of safe nursing behaviors in nursing students’ clinical practice: Importance–Performance Analysis (IPA), the borich needs assessment model, and the locus for focus model
Source: PLoS One. 2026 Mar 30;21(3):e0344741. doi: 10.1371/journal.pone.0344741 (PMC13035157; doi:10.1371/journal.pone.0344741)
Supplement: S2 Table — (DOCX) [file pone.0344741.s002.docx]

**Supplementary Table S2. Paired Comparisons of Importance and Performance Scores with and without Holm–Bonferroni Correction**

| Item | Gap | t | p-value | Rank | Holm-Bonferroni | Significance |
| --- | --- | --- | --- | --- | --- | --- |
| 1 | 0.21 | 5.06 | <0.001 | 1 | 0.002 | Yes |
| 2 | 0.12 | 4.38 | <0.001 | 2 | 0.002 | Yes |
| 3 | 0.19 | 5.38 | <0.001 | 3 | 0.002 | Yes |
| 4 | 0.21 | 5.06 | <0.001 | 4 | 0.002 | Yes |
| 5 | 0.14 | 4.07 | <0.001 | 5 | 0.002 | Yes |
| 6 | 0.46 | 6.91 | <0.001 | 6 | 0.002 | Yes |
| 7 | 0.33 | 7.11 | <0.001 | 7 | 0.002 | Yes |
| 8 | 0.18 | 4.1 | <0.001 | 8 | 0.002 | Yes |
| 9 | 0.11 | 4.23 | <0.001 | 9 | 0.003 | Yes |
| 14 | 0.24 | 5.31 | <0.001 | 10 | 0.003 | Yes |
| 15 | 0.29 | 7.01 | <0.001 | 11 | 0.003 | Yes |
| 16 | 0.48 | 7.74 | <0.001 | 12 | 0.003 | Yes |
| 17 | 0.26 | 6.39 | <0.001 | 13 | 0.003 | Yes |
| 18 | 0.35 | 5.81 | <0.001 | 14 | 0.003 | Yes |
| 19 | 0.6 | 9.03 | <0.001 | 15 | 0.004 | Yes |
| 20 | 0.25 | 4.84 | <0.001 | 16 | 0.004 | Yes |
| 21 | 0.32 | 6.87 | <0.001 | 17 | 0.004 | Yes |
| 22 | 0.15 | 4.08 | <0.001 | 18 | 0.005 | Yes |
| 23 | 0.19 | 5.43 | <0.001 | 19 | 0.005 | Yes |
| 24 | 0.17 | 4.59 | <0.001 | 20 | 0.006 | Yes |
| 26 | 0.23 | 4.46 | <0.001 | 21 | 0.006 | Yes |
| 27 | 0.19 | 4.13 | <0.001 | 22 | 0.007 | Yes |
| 28 | 0.22 | 4.38 | <0.001 | 23 | 0.008 | Yes |
| 25 | 0.14 | 3.55 | 0.001 | 24 | 0.010 | Yes |
| 12 | 0.13 | 2.89 | 0.004 | 25 | 0.013 | Yes |
| 13 | 0.05 | 2.89 | 0.004 | 26 | 0.017 | Yes |
| 10 | 0.02 | 1.35 | 0.181 | 27 | 0.025 | NO |
| 1 | 0.02 | 1.35 | 0.181 | 28 | 0.050 | NO |
