# Supplementary material for: Importance and implementation of safe nursing behaviors in nursing students’ clinical practice: Importance–Performance Analysis (IPA), the borich needs assessment model, and the locus for focus model
Source: PLoS One. 2026 Mar 30;21(3):e0344741. doi: 10.1371/journal.pone.0344741 (PMC13035157; doi:10.1371/journal.pone.0344741)
Supplement: S3 Table — (DOCX) [file pone.0344741.s003.docx]

**Supplementary Table S3. Integrated prioritization of safe nursing behavior items using Borich Needs Assessment, IPA, and Locus for Focus models**

| Final Priority Tier | Item | Borich rank  (Top-10) | Locus for Focus quadrant | IPA quadrant | Priority interpretation |
| --- | --- | --- | --- | --- | --- |
| First-tier priority | 7. Use of gown when biological exposure is possible | 5 | **HH** | CH | High importance and high discrepancy; immediate educational intervention required |
| First-tier priority | 21. Respect for personal dignity | 6 | **HH** | CH | High importance and high discrepancy; immediate educational intervention required |
| First-tier priority | 15. Maintain posture during ergonomic risks (e.g., transfers) | 7 | **HH** | CH | High-risk behavior with insufficient performance; urgent training needed |
| First-tier priority | 17. Education on chemical safety | 8 | **HH** | CH | Core knowledge deficit requiring structured education |
| Second-tier priority | 19. Know the location of spill kits | 1 | HL | LP | Large discrepancy despite lower perceived importance; potential institutional constraints. |
| Second-tier priority | 16. Use devices to reduce musculoskeletal load | 2 | HL | LP | Performance likely constrained by environmental resources; institutional support needed. |
| Second-tier priority | 6. Use of goggles when biological exposure is possible | 3 | HL | LP | Moderate discrepancy; supplementary PPE education recommended. |
| Second-tier priority | 18. Use PPE (apron, rubber gloves) for chemicals | 4 | HL | LP | Moderate discrepancy; situational training suggested. |
| Second-tier priority | 20. Education on psychological safety | 9 | HL | LP | Preventive education needed despite lower perceived importance. |
| Second-tier priority | 14. Education on musculoskeletal safety | 10 | HL | LP | Foundational education required for long-term injury prevention. |
