# Supplementary material for: Importance and implementation of safe nursing behaviors in nursing students’ clinical practice: Importance–Performance Analysis (IPA), the borich needs assessment model, and the locus for focus model
Source: PLoS One. 2026 Mar 30;21(3):e0344741. doi: 10.1371/journal.pone.0344741 (PMC13035157; doi:10.1371/journal.pone.0344741)
Supplement: S1 Appendix — (DOCX) [file pone.0344741.s004.docx]

**Appendix. Questionnaire on Safe Nursing Behaviors**

**Instructions**

The following items assess the perceived importance and performance of safe nursing behaviors during clinical practice.

Please indicate the level of importance and performance that you personally perceive for each item.

Responses are rated on a five-point Likert scale for both importance and performance.

Importance / Performance

- 1 = Strongly disagree
- 2 = Disagree
- 3 = Neutral
- 4 = Agree
- 5 = Strongly agree

| **Item** | **Importance** | | | | | **Performance** | | | | |
| --- | --- | --- | --- | --- | --- | --- | --- | --- | --- | --- |
| **I. Infection Prevention** | | | | | | | | | | |
| 1. Education and information on standard precautions | ① | ② | ③ | ④ | ⑤ | ① | ② | ③ | ④ | ⑤ |
| 2. Hand hygiene before and after glove use | ① | ② | ③ | ④ | ⑤ | ① | ② | ③ | ④ | ⑤ |
| 3. Hand hygiene after each patient contact | ① | ② | ③ | ④ | ⑤ | ① | ② | ③ | ④ | ⑤ |
| 4. Handwashing before leaving the patient room | ① | ② | ③ | ④ | ⑤ | ① | ② | ③ | ④ | ⑤ |
| 5. Use of gloves when biological exposure is possible | ① | ② | ③ | ④ | ⑤ | ① | ② | ③ | ④ | ⑤ |
| 6. Use of goggles when biological exposure is possible | ① | ② | ③ | ④ | ⑤ | ① | ② | ③ | ④ | ⑤ |
| 7. Use of gown when biological exposure is possible | ① | ② | ③ | ④ | ⑤ | ① | ② | ③ | ④ | ⑤ |
| 8. Avoid contamination when handling soiled linen | ① | ② | ③ | ④ | ⑤ | ① | ② | ③ | ④ | ⑤ |
| 9. Use disposable gloves for cleaning environment/equipment | ① | ② | ③ | ④ | ⑤ | ① | ② | ③ | ④ | ⑤ |
| 10. Dispose used ampoules/needles without recapping | ① | ② | ③ | ④ | ⑤ | ① | ② | ③ | ④ | ⑤ |
| 11. Dispose sharps into puncture-resistant containers | ① | ② | ③ | ④ | ⑤ | ① | ② | ③ | ④ | ⑤ |
| 12. Cover nose/mouth when coughing; hand hygiene after tissue disposal | ① | ② | ③ | ④ | ⑤ | ① | ② | ③ | ④ | ⑤ |
| 13. Wear a mask when having respiratory symptoms | ① | ② | ③ | ④ | ⑤ | ① | ② | ③ | ④ | ⑤ |
| **II. Musculoskeletal Injury Prevention** | | | | | | | | | | |
| 14. Education on musculoskeletal safety | ① | ② | ③ | ④ | ⑤ | ① | ② | ③ | ④ | ⑤ |
| 15. Maintain posture during ergonomic risks (e.g., transfers) | ① | ② | ③ | ④ | ⑤ | ① | ② | ③ | ④ | ⑤ |
| 16. Use devices to reduce musculoskeletal load | ① | ② | ③ | ④ | ⑤ | ① | ② | ③ | ④ | ⑤ |
| **III. Chemical Hazard Prevention** | | | | | | | | | | |
| 17. Education on chemical safety | ① | ② | ③ | ④ | ⑤ | ① | ② | ③ | ④ | ⑤ |
| 18. Use PPE (apron, rubber gloves) for chemicals | ① | ② | ③ | ④ | ⑤ | ① | ② | ③ | ④ | ⑤ |
| 19. Know the location of spill kits | ① | ② | ③ | ④ | ⑤ | ① | ② | ③ | ④ | ⑤ |
| **IV. Psychological Injury Prevention** | | | | | | | | | | |
| 20. Education on psychological safety | ① | ② | ③ | ④ | ⑤ | ① | ② | ③ | ④ | ⑤ |
| 21. Respect for personal dignity | ① | ② | ③ | ④ | ⑤ | ① | ② | ③ | ④ | ⑤ |
| 22. Positive engagement with peers | ① | ② | ③ | ④ | ⑤ | ① | ② | ③ | ④ | ⑤ |
| 23. Receive helpful feedback on practice | ① | ② | ③ | ④ | ⑤ | ① | ② | ③ | ④ | ⑤ |
| 24. No discrimination by age, gender, or school | ① | ② | ③ | ④ | ⑤ | ① | ② | ③ | ④ | ⑤ |
| 25. Appropriate response to verbal/physical violence | ① | ② | ③ | ④ | ⑤ | ① | ② | ③ | ④ | ⑤ |
| 26. Appropriate response to unwanted sexual attention | ① | ② | ③ | ④ | ⑤ | ① | ② | ③ | ④ | ⑤ |
| 27. Appropriate response to threats | ① | ② | ③ | ④ | ⑤ | ① | ② | ③ | ④ | ⑤ |
| 28. Appropriate response to humiliating behavior | ① | ② | ③ | ④ | ⑤ | ① | ② | ③ | ④ | ⑤ |
