# Supplementary material for: Importance and implementation of safe nursing behaviors in nursing students’ clinical practice: Importance–Performance Analysis (IPA), the borich needs assessment model, and the locus for focus model
Source: PLoS One. 2026 Mar 30;21(3):e0344741. doi: 10.1371/journal.pone.0344741 (PMC13035157; doi:10.1371/journal.pone.0344741)
Supplement: Attachment — Submitted filename: Reviewer Asmaa Kamal Ahmed.docx [file pone.0344741.s005.docx]

**Title:** Importance and implementation of safe nursing behaviors in nursing students' clinical practice: Importance–Performance Analysis (IPA), the Borich Needs Assessment model, and the Locus for Focus model

**Study Version:** 30/11/ 2025

Reviewer: Dr. Asmaa Kamal Ahmed

**Reviewer's report:**

Dear Authors,

The paper reports a cross-sectional survey of 160 nursing students from two South Korean universities to assess the perceived importance and self-reported performance of 29 safe nursing behaviors across four domains (infection, musculoskeletal, chemical, psychological). Using Importance–Performance Analysis (IPA), the Borich Needs Assessment model, and the Locus for Focus model, the authors prioritize items for educational intervention, highlighting gaps in PPE use under biological exposure, ergonomic practices, chemical safety knowledge (e.g., spill kits), and psychological safety (respect for dignity). The study aims to inform targeted curriculum design for nursing safety education.

**Specific Comments**

Strengths

**Technical novelty and innovation**

- Combines three complementary prioritization frameworks (IPA, Borich, Locus for Focus) to triangulate educational needs, mitigating the known limitations of each method in isolation.
- Develops a structured 29-item instrument with expert content validation and reports strong internal consistency (α = 0.90).

**Experimental rigor and validation**

- Adequate sample size with a priori power analysis; IRB approval and informed consent procedures are clearly described.
- Employs appropriate statistical tools (paired t-tests, IPA, Borich, Locus for Focus) to identify gaps between importance and performance.

**Clarity of presentation**

- Constructs a coherent narrative from risk context to measurement to prioritization, producing actionable categories for curriculum design.
- Presents item-level results and domain-level insights that translate naturally into educational interventions.

**Significance of contributions**

- Addresses a high-impact area—nursing student and patient safety—within a defined policy context (Korean Patient Safety Act).
- Offers pragmatic priorities (e.g., chemical spill preparedness, ergonomic supports) that can directly inform course content and practicum policies.

**Weaknesses and Suggestions for Improvement**

- Instrument development: Content validity via expert panel and reporting of overall α = 0.90 are strengths; however, a factor-analytic study (EFA/CFA) is needed to confirm dimensionality and item loadings across the four domains. Report domain-level α and separate reliability for importance and performance ratings.
- Measurement scaling: Clarify and align scale anchors for importance (“not important” to “very important”) versus performance (“strongly disagree” to “strongly agree,” or similar). If anchors differed, report them explicitly and justify comparability.
- Statistical testing: With 29 paired comparisons, apply a multiple-comparison control (e.g., Holm-Bonferroni) or at least report adjusted p-values; also report effect sizes (e.g., Cohen’s d for paired samples) and 95% confidence intervals for all gaps.
- IPA specification: The choice of grand-mean crosshairs can be arbitrary; consider sensitivity analyses (e.g., scale midpoints, percentile crosshairs, diagonally shifted IPA) and/or derived-importance approaches. Clearly define the crosshair values used (you report means of 3.93 and 3.70; ensure consistency across text, figure, and tables).
- Locus for Focus and Borich: Provide formulas explicitly; for Locus for Focus, define discrepancy (importance − performance) and the thresholding at grand means. For Borich, specify the exact computation and verify ranking reproducibility (share code or spreadsheet if possible).

**Experimental evaluation assessment**

- Sampling and generalizability: Acknowledge the limits of convenience sampling from two universities; include demographic and practicum-context descriptors (e.g., practice settings) that may affect safety behaviors. Consider stratified analyses by year, prior training, and practicum type.
- Data integrity: Resolve inconsistencies in Table 2 (e.g., mask item gap 0.50 vs means 3.98–3.93, and the interpretation of Quadrants III/IV in Section 3.3). Ensure all item-level statistics reconcile with quadrant assignments and model rankings.
- Additional analyses: Explore whether prior safety education (reported by 88.1%) moderates gaps; analyze dose-response (e.g., number/hours of training) if available. Consider regression models to identify predictors of larger gaps.
- Reporting: Provide the complete instrument (English and Korean) in supplementary materials, including translation/back-translation procedures if applicable.

**Comparison with related work (using the summaries provided)**

- Musculoskeletal risks: Your prioritization of ergonomic posture and assistive device use aligns with broader occupational health evidence that “working posture” is a severe MSD factor (Related Work: 2312.11517) and that risk classification/monitoring tools (e.g., EMG/ML, 2109.15036) can augment training. Briefly situate your findings within these trends and discuss potential technology-enabled interventions.
- Training modalities: The interest in simulation aligns with recommendations to include more empathetic, realistic scenarios in training for caregiving contexts (2405.08948). Consider discussing how prioritized items (e.g., chemical spill response) could be embedded into simulation or VR-based modules.
- IPA methodology: Other domains using IPA (2301.01278; 2103.09049) highlight choices around crosshairs and the value of communicating quadrant rationale; referencing these can help contextualize your methodological choices and motivate sensitivity checks.

**Discussion of broader impact and significance**

- The identified priorities—PPE under biological exposure, ergonomic practices, chemical spill preparedness, and psychological safety—map directly to curricular modules and practicum policies. Provide concrete curricular implications (e.g., scenario-based simulations for spill response; structured de-escalation and dignity/respect training; checklists and device availability for lifting/transfers).
- Consider institutional levers (policy, supervision, resource provision) alongside student education, especially for items where performance may be constrained by access (e.g., spill kits, ergonomics devices).
- Address how targeting these priorities may advance both student safety and patient safety; propose metrics for follow-up evaluation (e.g., incident reports, near-miss tracking, OSH compliance audits).

**Questions for Authors:**

1- Please clarify the measurement scales and anchors used for importance vs performance. Were the anchors identical or tailored per construct?
2- In Table 2, for “Wear a mask when having respiratory symptoms,” the reported gap (0.50 ± 0.22) appears inconsistent with the means (3.98 vs 3.93). Is this a typographical error? If so, please correct and update related statistics (t, p, Borich rank).
3- In Section 3.3, Quadrants III and IV are described in ways that conflict with standard IPA definitions and your own earlier definitions. Could you correct the quadrant descriptions and verify the item assignments accordingly?
4- How were missing data handled (if any) at the item level? Please report item-wise response rates and any imputation procedures.
5- Did you compute reliability (Cronbach’s alpha) separately for importance items and performance items, and for each domain? If so, please report them; if not, could you add these analyses?
6- Was any factor analysis (EFA or CFA) conducted to validate the four-domain structure? If not, could you provide at least an EFA to support the latent structure?
7- Given 29 paired comparisons, did you adjust for multiple testing? If not, how robust are your conclusions under a correction (e.g., Holm-Bonferroni)?
8- The domain-level “perceived performance levels” in Table 1 (e.g., 4.75 for infection prevention) differ notably from the item-level means (~3.7–3.98). How were these domain-level scores derived? Are they separate global items, and if so, how should readers interpret both?
9- Could you provide a single integrated table listing: (a) the Borich top-10 items, (b) Locus for Focus quadrant placement, (c) IPA quadrant placement, and (d) the final prioritized list with clear first/second-tier priorities?
10- For items like “Know the location of spill kits” and “Use devices to reduce musculoskeletal load,” to what extent do resource availability and institutional policies constrain performance? How might you disentangle education gaps from environmental constraints?
11- Did prior safety education (88.1% reported) associate with smaller gaps? If you tested this, please share the results; if not, could you analyze it?
12- Was the instrument administered in Korean? If so, please describe translation/back-translation and include the final instrument in supplementary materials.

**Recommendation**

This is a timely and practically oriented study that addresses an important gap: identifying concrete priorities for improving nursing students’ safety behaviors in clinical practicicum. The triangulation across IPA, Borich, and Locus for Focus is a strong design choice, and the findings translate well into actionable educational targets (e.g., PPE use, ergonomics, chemical spill readiness, psychological safety). However, several issues require careful revision before the results can be relied upon for curricular decisions: (1) inconsistencies and likely errors in Table 2 and quadrant descriptions, (2) incomplete psychometric validation and lack of separate reliability reporting for importance vs performance, (3) absence of multiple-comparison correction and effect size reporting, and (4) limited clarity on the relation between domain-level and item-level scores. Addressing these points, alongside enhanced methodological transparency (e.g., crosshair choices, sensitivity analyses) and a consolidated priority list would substantially strengthen the paper. With these revisions, the work would offer solid, evidence-based guidance to nursing education programs and contribute useful methodology for prioritizing safety competencies. I recommend major revision.
