# Supplementary material for: Importance and implementation of safe nursing behaviors in nursing students’ clinical practice: Importance–Performance Analysis (IPA), the borich needs assessment model, and the locus for focus model
Source: PLoS One. 2026 Mar 30;21(3):e0344741. doi: 10.1371/journal.pone.0344741 (PMC13035157; doi:10.1371/journal.pone.0344741)
Supplement: Attachment — Submitted filename: Response to Reviewers3_R(1)_PONE-D-25-53140R1.docx [file pone.0344741.s006.docx]

Manuscript ID: PONE-D-25-53140R1

Type of manuscript: Article

Title: Importance and implementation of safe nursing behaviors in nursing students' clinical practice: Importance–Performance Analysis (IPA), the Borich Needs Assessment model, and the Locus for Focus model

Thank you for reviewing this study. We have attempted to address the reviewer's comments and improve the manuscript. We have included point-by-point responses to each comment in this resubmission. The detailed responses to each comment are as follows:

**Response to Reviewer 3 Comments.**

1. The manuscript is well written and clearly presented.
2. The study is interesting and highly relevant to the training of nursing students in practical settings, particularly with respect to their safety and that of the patient.

3. However, Figures 1 and 2 require revision, as their content does not fully align with the narrative. To enhance reader comprehension and ensure consistency with the text, the quadrant boundaries should include appropriate titles or labels that clearly indicate the position of each quadrant.

**Reply:**

- We appreciate the reviewer’s helpful comment. To improve clarity and ensure consistency between the figures and the text, Figures 1 and 2 have been revised to include clear titles and labels for each quadrant, explicitly indicating their conceptual meanings (e.g., Keep up the Good Work, Concentrate Here, Low Priority, and Possible Overkill for the IPA; and High Discrepancy/High Importance, High Discrepancy/Low Importance, etc., for the Locus for Focus model).

In addition, the figure legends have been revised to clearly define all quadrant abbreviations and reference axes, allowing readers to more easily interpret the figures without ambiguity. The placement of items within each quadrant was also carefully reviewed to ensure full alignment with the descriptions in the Results section. These revisions enhance visual clarity and strengthen consistency between the figures, legends, and the narrative.
